# Adsorption Property and Mechanism of Oxytetracycline onto Willow Residues

**DOI:** 10.3390/ijerph15010008

**Published:** 2017-12-22

**Authors:** Di Wang, Haiyang Xu, Shengke Yang, Wenke Wang, Yanhua Wang

**Affiliations:** 1 Key Laboratory of Subsurface Hydrology and Ecological Effects in Arid Region, Ministry of Education, Chang’an University, Xi’an 710054, China; wd4591@126.com (D.W.); wenkew@chd.edu.cn (W.W.); 2 School of Environmental Science and Engineering, Chang’an University, Xi’an 710054, China; 3 College of Natural Resources and Environment, Northwest A&F University, Yangling 712100, China; 4 Liaoning Zhongwang Group Co., Ltd., Liaoyang 111003, China; 13386793382@163.com; 5 School of Geography and Tourism, Shaanxi Normal University, Xi’an 710054, China

**Keywords:** PPCPs, oxytetracycline, willow residues, adsorption

## Abstract

To elucidate the adsorption property and the mechanism of plant residues to reduce oxytetracycline (OTC), the adsorption of OTC onto raw willow roots (WR-R), stems (WS-R), leaves (WL-R), and adsorption onto desugared willow roots (WR-D), stems (WS-D), and leaves (WL-D) were investigated. The structural characterization was analyzed by scanning electron microscopy, Fourier-transform infrared spectra, and an elemental analyzer. OTC adsorption onto the different tissues of willow residues was compared and correlated with their structures. The adsorption kinetics of OTC onto willow residues was found to follow the pseudo-first-order model. The isothermal adsorption process of OTC onto the different tissues of willow residues followed the Langmuir and Freundlich model and the process was also a spontaneous endothermic reaction, which was mainly physical adsorption. After the willow residues were desugared, the polarity decreased and the aromaticity increased, which explained why the adsorption amounts of the desugared willow residues were higher than those of the unmodified residues. These observations suggest that the raw and modified willow residues have great potential as adsorbents to remove organic pollutants.

## 1. Introduction

Antibiotics are widely and frequently used in numerous kinds of Pharmaceuticals and Personal Care Products (PPCPs). Recently, there has been a lot of concern about the presence of antibiotics in water and sediments due to their toxicity, their disturbance to the ecosystem function, and their poor biodegradability [1]. Oxytetracycline (OTC) is one of the most common antibiotics and it has proved to be difficult to biodegrade. Nowadays, residual OTC has already been detected in many countries and areas. In British soil, which was applied with manure, the concentration of OTC was as high as 1691 μg/kg [2]. In northern Turkey, OTC was detected in all manured agricultural soil with a maximum concentration of 500 μg/kg [3]. In China, the contents of OTC in sediment and in aquaculture field soil were as high as 200 mg/kg and 285 mg/kg, respectively [4,5]. Thus, the residues of OTC in the environment have been confirmed to be a global issue. There are many methods available for antibiotic removal, including adsorption [6], oxidation [7], photochemical degradation [8], electrochemical treatments [9], and membrane filtration [10]. Among these methods, adsorption is considered to be one of the most effective methods for removing OTC with a low concentration. Some scholars have studied the adsorption of OTC with different materials in recent years. Huang et al. [11] studied the characterization of activated carbons that were prepared by microwave and conventional heating methods, and their application in the removal of OTC. Wang et al. [12] focused on the adsorption of OTC onto three kinds of commercial resins. Liu et al. [13] found that the modified corn straw was an effective adsorbent in removing OTC from wastewater. It can be seen that the adsorption of OTC onto different materials has been studied. However, the adsorption of OTC onto plant residues has rarely been the focus.

Plant residues as a natural organic matter, are considered as one of the most abundant, cheap, and renewable resources. Furthermore, plant residues are also a common biological adsorbent. Boving et al. [14] showed that aspen wood was an effective adsorbent for the removal of polycyclic aromatic hydrocarbons (PAHs). Chen et al. [15] reported that PAHs were effectively removed from wastewater by using wood chips, ryegrass roots, orange peels, bamboo leaves, and pine needles. Xi et al. [16] found that raw and modified plant residues both have great potential as effective, low-cost biosorbents for the removal of naphthalene, acenaphthene, phenanthrene, and pyrene in an aqueous solution. Lin et al. [17] found that raw and brewed tea leaves have high adsorption capacities for the removal of phenanthrene. So far, studies on the adsorption of plant residues have mainly concentrated on hydrophobic organic pollutants, and the roles of plant residues in the removal of hydrophilic organic pollutants require further study.

The main objectives of this study are given as follows: (1) to investigate the adsorption kinetics, isotherms, and thermodynamic properties involved in OTC-willow residues surface reaction; (2) to evaluate the structural characterization of different tissues of willow residues by the use of scanning electron microscopy (SEM), Fourier-transform infrared (FTIR) spectra, and an elemental analyzer. For this purpose, OTC was chosen as a representative of typical hydrophilic organic pollutants, and willow residues were chosen as the model plant residues. Willow residues, including willow roots (WR), willow stems (WS), and willow leaves (WL) were selected and modified via acid hydrolysis to obtain desugared samples. The purposes of modification are also listed as follows: (1) to remove polysaccharides from willow residues; (2) to simulate the accelerated decay process of willow residues.

## 2. Materials and Methods

### 2.1. Chemical Reagents

Oxytetracycline was purchased from Boston Biomedical Inc. (Cambridge, MA, USA) with a United States Patent (USP) grade. The molecular formula of OTC is C_22_H_24_N_2_O_9_, and its molecular weight is 460.44. Methanol in its flowing phase was purchased from the Waters Company (Milford, MA, USA) with a High Performance Liquid Chromatography (HPLC) grade. The other reagents were all in an analytical grade.

### 2.2. Preparation of Willow Residues

Willow residues were collected from Chanba Ecological District, Xi’an, China (109°02′41″ E, 34°19′36″ N) in July 2015. These samples were washed with deionized distilled water to remove dust and were then oven-dried for 12 h at 60 °C, ground with a pulverizer, and were sieved to less than 0.25 mm, yielding raw samples. The raw samples were desugared by acid modification, using a reported method [18]. Acid modification, conducted in a 6 mol/L HCl solution with refluxing for 6 h at 100 °C was used to eliminate the polysaccharide component from raw willow roots (WR-R), raw willow stems (WS-R), and raw willow leaves (WL-R), and produced desugared willow roots (WR-D), desugared willow stems (WS-D), and desugared willow leaves (WL-D), respectively. The sugar content of the raw willow residues was calculated from the yields of the modified fractions. All desugared willow residues were separated from the acidic solution by filtration and then were washed with deionized distilled water to adjust these residues to neutral conditions and to remove any dissolved organic matter that had adsorbed onto the residues. All raw and desugared willow residues were oven-dried at 60 °C, ground, and sieved to less than 0.25 mm before analysis and adsorption experiments.

### 2.3. Characterization of Willow Residues

The raw and desugared willow residues were examined with a KYKY-2800B scanning electron microscope (SEM) (KYKY, Beijing, China) under high vacuum conditions and at an accelerating voltage of 25 kV, in order to observe the surface morphology of the samples. Prior to testing with the SEM, each sample was coated with a thin, electrically conductive gold film. The Fourier-transform infrared (FTIR) spectra of the raw and desugared willow residues were recorded in the 4000–400 cm^−1^ region for a KBr pellet (to ensure 20–80% transmittance rate) by a Nicolet FTIR spectra (model 6700, Thermo Scientific, Waltham, MA, USA) with a resolution of 4.0 cm^−1^. Elemental (C, H, O, N) analysis of the raw and desugared willow residues was conducted by using a Vario EL cube elemental analyzer (Elementar, Frankfurt, Germany). The H/C, (N + O)/C, and O/C atomic ratios of willow residues were calculated to evaluate their aromaticity, polarity, and hydrophilicity, respectively.

### 2.4. Detection Method of OTC in Solution

A Waters ACQUITY Ultra Performance Liquid Chromatography (UPLC) H-Class (Waters, Milford, MA, USA) with an ultraviolet/visible spectrophotometer detector and a C18 1.7 μm 2.1 × 150 mm column were used for the quantification of OTC in the solution, under the optimized conditions as follows: methanol/water = 60:40 as flowing phase, at a flow rate of 0.2 mL/min, with a column temperature of 60 °C ± 0.1 °C, a sample temperature of 10 °C ± 0.1 °C, an injection volume of 5 μL, a detected wavelength of 260 nm, and a retention time of 1.960 min.

### 2.5. Adsorption Procedure

#### 2.5.1. Adsorption Kinetics

To conduct the adsorption kinetic experiments, the batch studies were carried out by weighing 0.10 g of willow residues and adding it into a 50 mL graduated centrifuge tube. After 30 mL of the OTC solution was added into the graduated centrifuge tube, the mixture was placed on a rotating shaker and was agitated end-over-end in the dark at 150 r/min and at 25 °C for 90 h. The initial concentration of OTC was 10.00 mg/L and the aqueous samples were separated at predetermined time intervals using filter membranes of 0.22 μm. The concentrations of OTC were analyzed by UPLC. All of the adsorption experiments were performed in duplicate, and the experimental details are given in the related figures and tables. Blanks containing no OTC were analyzed and the loss (generally quite low) was considered. The uptake of OTC at time t, Q_t_ (mg/kg), was calculated using the following equation:

(1) $$Q_{t}=\frac{V(C_{0}-C_{t})}{m}$$

where C_0_ and C_t_ are the initial concentration of OTC (mg/L) in the solution and the concentration at time (t), respectively, V is the volume of the solution (L), and m is the weight of the willow residues (g).

#### 2.5.2. Adsorption Isotherm

For the adsorption isotherm studies, solutions with different initial concentrations were added, which ranged from 5.0 to 30.0 mg/L (5.0, 10.0, 15.0, 20.0, and 30.0 mg/L, pH = 7.0). The equilibrium time of the raw willow residues was set as 12 h, while the equilibrium time of the desugared willow residues was set as 24 h. All of the equilibrium times were enough according to the adsorption kinetic studies. The aqueous samples were separated by using filter membranes of 0.22 μm. The concentrations of OTC were analyzed by UPLC. Each initial concentration had two replicate samples. Blanks containing no OTC were analyzed and the loss (generally quite low) was considered. The uptake of OTC at equilibrium, Q_e_ (mg/kg), was calculated by using the following equation:

(2) $$Q_{e}=\frac{V(C_{0}-C_{e})}{m}$$

where C_e_ is the equilibrium concentration of OTC (mg/L) in the solution.

#### 2.5.3. Thermodynamics

For the thermodynamics studies, the initial concentration ranged from 5.0 to 30.0 mg/L (5.0, 10.0, 15.0, 20.0, and 30.0 mg/L, pH = 7.0) at 25 °C (298 K), 35 °C (308 K), and at 45 °C (318 K). After equilibrium time, the aqueous samples were separated using filter membranes of 0.22 μm. The concentrations of OTC were analyzed by UPLC. Blanks containing no OTC were analyzed and the loss (generally quite low) was considered.

## 3. Results and Discussion

### 3.1. Adsorption Kinetics

**Figure 1:**
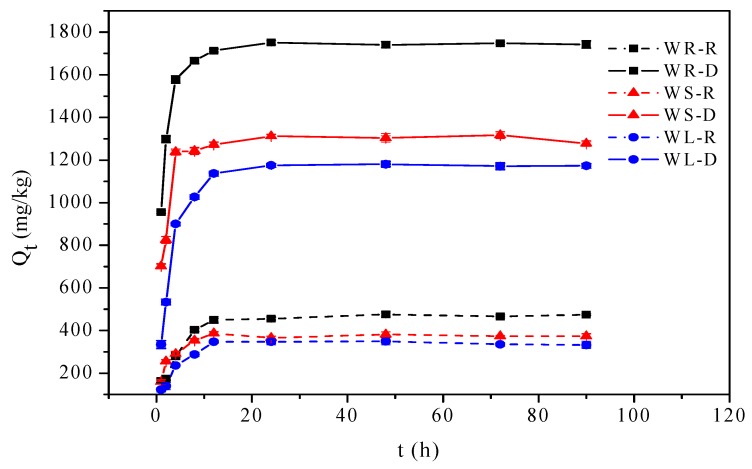
Effect of contact time for oxytetracycline (OTC) adsorption onto willow residues.

The adsorption of OTC onto willow residues as a function of contact time at solution pH = 7.0 is shown in Figure 1. The adsorption processes of the raw willow residues were mostly completed within the first 8 h of reaction and then became more gradual until equilibrium was reached within 12 h. Meanwhile, the adsorption processes of the desugared willow residues were mostly completed within the first 12 h of reaction and equilibrium was reached within 24 h. It was possible that at the beginning of adsorption, enough adsorption sites existed on the surface of the willow samples, which was beneficial to the adsorption of OTC. With increasing contact time, adsorption sites on the surface of willow samples were close to saturation, which caused the adsorption of OTC transfer from the surface into voids of the willow samples.

Two widely used kinetic models, pseudo-first-order and pseudo-second-order kinetic models, were employed to interpret the kinetics results. The linearized form of the pseudo-first-order kinetic model is given as follows [19]:

(3) $${\ln\;(Q}_{e}-Q_{t})={\mathrm{lnQ}}_{e}-k_{1}t$$

where Q_e_ (mg/kg) and Q_t_ (mg/kg) are the OTC adsorption amounts onto willow residues at equilibrium and at any time t (h), respectively. k_1_ (1/h) is the rate constant of the pseudo-first-order kinetic model. The results are given in Table 1. The linearized form of the pseudo-second-order kinetic model is given as follows [19]:

(4) $$\frac{t}{Q_{t}}=\frac{1}{k_{2}Q_{e}^{2}}+\frac{t}{Q_{e}}$$

where k_2_ (kg/mg·h) is the rate constant of the pseudo-second-order kinetic model. The values of the kinetic model parameters (Q_e_, k_1,_ and k_2_) along with the corresponding correlation coefficients can be derived using Origin Pro 8.5. The regression results are listed in Table 1.

The pseudo-first-order and the pseudo-second-order kinetic models were both well followed; however, the fit (R^2^) of the pseudo-first-order kinetic model was better, and the calculated Q_e,cal_ values were in agreement with the experimental results (Q_e,exp_), suggesting the applicability of the pseudo-first-order kinetic model in describing the adsorption kinetics data of OTC onto willow residues.

### 3.2. Adsorption Isotherms

The adsorption isotherms of OTC onto willow residues were studied at 25 °C (298 K, pH = 7.0). Two-parameter isotherm models (Langmuir and Freundlich) were used to fit the experimental data which are shown in Table 2. The linearized form of the Langmuir isotherm model can be written as follows [20,21]:

(5) $$\frac{C_{e}}{Q_{e}}=\frac{1}{Q_{m}K_{L}}+\frac{C_{e}}{Q_{m}}$$

where C_e_ is the equilibrium concentration of OTC (mg/L) in the solution. Q_e_ (mg/kg) is the amount of OTC adsorption onto willow residues. Q_m_ (mg/kg) is the Langmuir adsorption constant, representing the maximum adsorption amounts. The practical limiting adsorption amounts, when the surface of the willow residues is completely covered with OTC, are represented by Q_m_. K_L_ is the Langmuir adsorption constant, which is related to the affinity of binding sites and hence, the adsorption bonding energy. The essential quality of the Langmuir isotherm can be measured by calculating a dimensionless constant, referred to as the separation factor, R_L_, which is defined as follows [20,21]:

(6) $$R_{L}=\frac{1}{1+K_{L}C_{0}}$$

where C_0_ (mg/L) is the initial concentration of OTC in the solution. The R_L_ has the following possibilities: 0 < R_L_ < 1 for a favorable adsorption; R_L_ > 1 for an unfavorable adsorption; R_L_ = 1 for a linear adsorption; R_L_ = 0 for an irreversible adsorption.

The linearized form of the Freundlich isotherm model can be written as follows [20,21]:

(7) $$\ln Q_{e}=\ln K_{F}+\frac{1}{n}\ln C_{e}$$

where K_F_ ((mg/kg) (L/mg)^1/n^) and “n” are the Freundlich constants which are related to the adsorption amounts and the adsorption intensity, respectively.

The parameters of the Langmuir and Freundlich isotherm models for OTC adsorption onto willow residues, along with the values of the determination coefficient R^2^ are given in Table 2. The values of R^2^ for the Langmuir and Freundlich isotherm models were both high. They proved that the Langmuir and Freundlich isotherm models were both suitable in describing the adsorption behavior of OTC onto willow residues.

For the Langmuir isotherm model, the R_L_ for OTC adsorption onto WR-R, WS-R, and WL-R ranged from 0.93–0.99, 0.79–0.96, and from 0.86–0.97, respectively; the R_L_ for OTC adsorption onto WR-D, WS-D, and WL-D ranged from 0.74–0.95, 0.70–0.93, and from 0.64–0.91, respectively. All of the ranges of R_L_ are lying between 0 and 1, indicating a favorable adsorption [22].

For the Freundlich isotherm model, K_F_ is indicative of the adsorption amounts of willow residues. The greater the K_F_ value, the greater the adsorption amounts. It showed that desugarization can enhance the adsorption of OTC onto willow residues. Because “n” is related to the adsorption intensity, and the value of “n” for all of the willow residues is larger than 1, which indicates a favourable nature of adsorption of OTC onto willow residues [23].

### 3.3. Thermodynamics

The adsorption of OTC onto willow residues was studied as a function of temperature (298 K, 308 K and 318 K, pH = 7.0). The obtained results are listed in Table 3. The values of K_L_ for the Langmuir isotherm at 298 K, 308 K, and at 318 K were used to calculate thermodynamic parameters such as the Gibbs free energy change (ΔG), the enthalpy change (ΔH), and the entropy change (ΔS) using the following equations [21]:

(8) $$\Delta G=-\mathrm{RTln}K_{L}$$

(9) ΔG=ΔH−TΔS

where K_L_ (L/mol) is the Langmuir constant, R (8.314 J/mol·K) is the gas constant, and T (K) is the absolute temperature. The obtained values of the thermodynamic parameters for the OTC adsorption onto willow residues are given in Table 3. The results showed that the amounts of OTC adsorption for the willow residues increased with increasing temperature, which indicated that the adsorption of OTC onto willow residues is favored at a higher temperature. The negative values of ΔG suggest the feasibility of the adsorption of OTC onto willow residues and the spontaneous nature of the adsorption process [24]. The positive values of ΔH indicated that the adsorption process is endothermic in nature [25]. The positive values of ΔS showed an increase in randomness at the solid/liquid interface during the adsorption process [26].

### 3.4. Analysis of the Adsorption Mechanism of OTC Adsorption onto Willow Residues

To better understand the adsorption mechanism of OTC adsorption onto willow residues, the structures of the different tissues of raw and desugared willow residues were studied using various methods such as SEM, FTIR spectra analysis, and Elemental analysis.

#### 3.4.1. SEM Analysis

**Figure 2:**
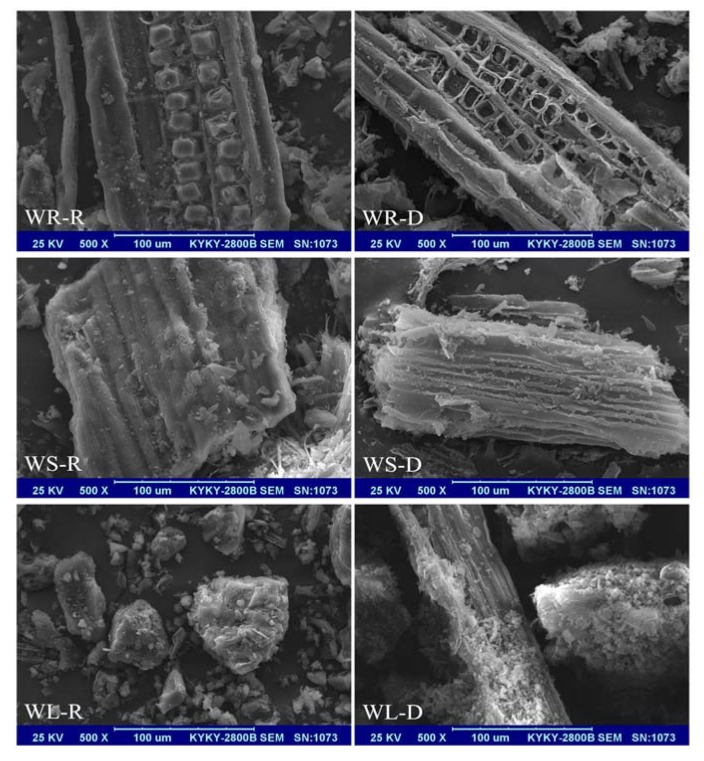
SEM images of the raw and desugared willow residues.

The surface morphologies of the raw and the desugared willow residues are shown in Figure 2. It can be seen that the surface morphologies of WR-R, WS-R, and WL-R were abundant, and the availability of pores and the internal surface are clearly displayed in the image. Compared with the raw willow residues, the desugared samples showed a larger specific surface area and the adsorption sites increased. After being desugared, the skeleton structure still existed in the samples of WR-D, WS-D, and WL-D. The surface morphologies of the desugared willow residues were more uneven and rougher than those of the raw willow residues.

#### 3.4.2. FTIR Spectra Analysis

**Figure 3:**
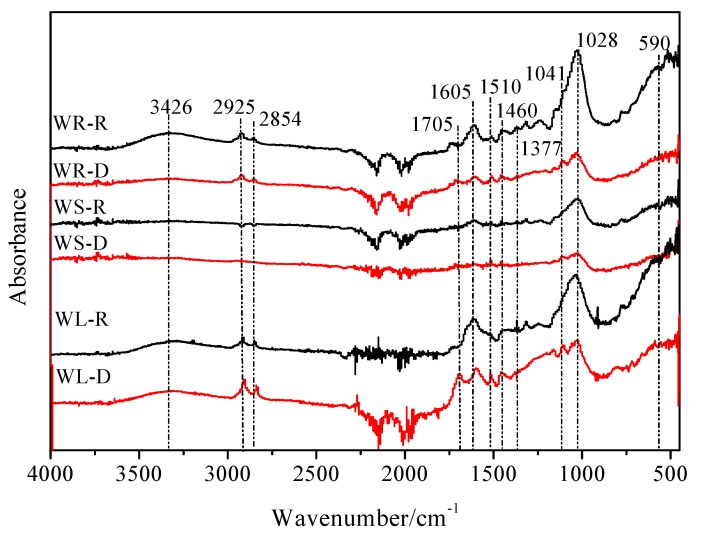
FTIR spectra of the raw and desugared willow residues.

The FTIR spectra between 4000–400 cm^−1^ for the willow residues are shown in Figure 3. The peaks at 1028 and 1041 cm^−1^ are assigned to C–O–C stretching of polysaccharides. The peak at 1510 cm^−1^ represents the C=C ring stretching vibration of lignin and the band at 1605 cm^−1^ is assigned to the aromatic C=C and C=O [18]. The band at 1705 cm^−1^ is assigned to C=O and C–O stretching vibrations of ester bonds. The peaks at 1377, 1460, 2854, and 2925 cm^−1^ are assigned mainly to CH_2_ units [27]. The band at 3426 cm^−1^ represents the stretching vibration of hydroxyl groups (–OH).

For the raw willow residues, the peaks at 1028 and 1041 cm^−1^ represent the dominant role of carbohydrate functional groups (C–O–C). For the desugared willow residues, the peaks at 1510, 1605, and 1705 cm^−1^ represent the dominant role of the functional group of C=C, C=O, and –COOH. The peaks of –OH (3426 cm^−1^) and C–O–C (1041 and 1028 cm^−1^) of carbohydrates were sharply reduced and the CH_2_ bands (2925, 2854 and 1460 cm^−1^) became stronger after removing the polysaccharides. At the same time, after removing the polysaccharides, the aromatic fraction of the willow residues was exposed, and the adsorption amounts increased greatly, compared to the raw samples, which was consistent with the dynamic experimental results [18,28].

#### 3.4.3. Elemental Analysis

The elemental compositions of the different tissues of raw and desugared willow residues are presented in Table 4. The six samples contained a high content of C, H, and O, and a low content of N. Compared with the raw willow residues, the organic carbon content of the desugared willow residues increased with the decrease in oxygen content; correspondingly, the hydrophilic indexes decreased, the polarity indexes dropped sharply, and aromaticity indexes enhanced notably due to the removal of the polysaccharides (polar components). WL-R, with the highest sugar content, saw the largest change in polarity and aromaticity after removing the polysaccharides: the polarity index decreased from 0.77 to 0.44, and the aromaticity decreased from 1.70 to 1.35; WR-R, with the lowest sugar content, had the smallest change in polarity and aromaticity after removing the polysaccharides: the polarity index decreased from 0.82 to 0.65, and the aromaticity decreased from 1.65 to 1.39.

Partition coefficient (K_d_) is used to describe the adsorption efficiency for organic pollutants. The K_d_ for OTC adsorption onto WR-R, WS-R, and WL-R ranged from 49.42–55.01, 38.88–49.38, and 37.66–46.20, respectively; the K_d_ for OTC adsorption onto WR-D, WS-D, and WL-D ranged from 196.42–234.10, 129.36–176.84, and 124.34–153.59, respectively. The values of K_d_ increased after removing the polysaccharides, indicating that acid modification was an effective modification method to enhance adsorption amounts of adsorbents.

**Figure 4:**
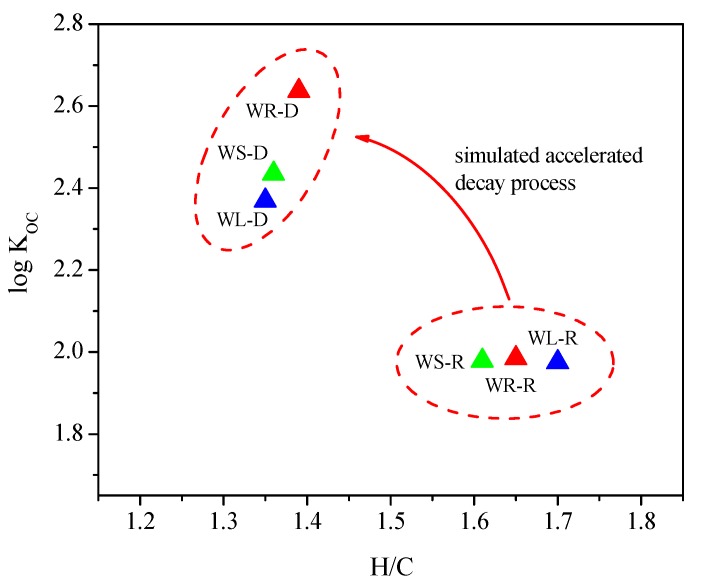
Relationship between the aromaticity index (H/C) and log K_oc_ of willow residues in accelerated decay process.

K_oc_ is the carbon normalized adsorption coefficient, and the relationship between the aromaticity index (H/C) and log K_oc_ of willow residues in accelerated decay process is shown in Figure 4. It can be seen from Figure 4 that, with the increase of aromaticity (H/C), the values of log K_oc_ were increasing gradually; correspondingly, the adsorption of OTC had significantly increased. This indicated that the content of organic carbon and aromaticity played a key role in the adsorption of OTC onto willow residues and acid modification treatment can improve the adsorption amounts of OTC onto willow residues.

## 4. Conclusions

The present study shows that the different tissues of raw and desugared willow residues both have great potential as effective adsorbents for OTC removal. Based on the results of this study, we can conclude the following.

The adsorption of OTC onto raw willow residues reached equilibrium in about 12 h, while desugared willow residues reached equilibrium in about 24 h. The adsorption kinetics of OTC onto willow residues was found to follow the pseudo-first-order model.

The isothermal adsorption process of OTC onto the different tissues of willow residues followed the Langmuir and Freundlich model and the process was a spontaneous endothermic reaction which was mainly physical adsorption.

The elemental analysis, the scanning electron microscope, and the Fourier-transform infrared spectrum showed that the content of organic carbon in the willow residues after the removal of sugar was increased, the polarity was decreased, the aromaticity was increased, and the specific surface area was increased, which from the microscopic angle, further illustrated the reason why the adsorption amounts of willow residues were improved after desugarization.

## Figures and Tables

**Table 1:** Pseudo-first-order and pseudo-second-order rate constants for OTC adsorption onto willow residues.

Samples	Q_e,exp_	Pseudo-First-Order Model			Pseudo-Second-Order Model		
		R^2^	k_1_	Q_e,cal_	R^2^	k_2_	Q_e,cal_
	(mg/kg)		(1/h)	(mg/kg)		(kg/mg·h)	(mg/kg)
Raw willow roots (WR-R)	464.32	0.9740	0.2409	467.42	0.9700	0.0008	486.91
Desugared willow roots (WR-D)	1745.84	0.9853	0.7649	1727.43	0.9819	0.0007	1793.56
Raw willow stems (WS-R)	376.58	0.9564	0.5317	374.62	0.9098	0.0024	388.55
Desugared willow stems (WS-D)	1302.04	0.9591	0.7166	1289.20	0.9483	0.0009	1337.26
Raw willow leaves (WL-R)	342.69	0.9686	0.2909	344.65	0.9362	0.0012	370.24
Desugared willow leaves (WL-D)	1175.11	0.9881	0.3368	1163.17	0.9436	0.0004	1283.42

**Table 2:** Isotherm parameters for OTC adsorption onto willow residues.

Samples	Langmuir Model				Freundlich Model		
	Q_m_	K_L_	R^2^	R_L_	K_F_	n	R^2^
	(mg/kg)	(L/mg)			(mg/kg) (L/mg)^1/n^		
WR-R	20,223.57	0.0027	0.9912	(0.93, 0.99)	56.43	1.03	0.9906
WR-D	21,932.29	0.0116	0.9844	(0.74, 0.95)	301.81	1.15	0.9796
WS-R	5936.72	0.0087	0.9624	(0.79, 0.96)	21.04	1.10	0.9544
WS-D	12,188.92	0.0146	0.9967	(0.70, 0.93)	233.00	1.23	0.9965
WL-R	8216.63	0.0055	0.9687	(0.86, 0.97)	16.17	1.06	0.9657
WL-D	9780.79	0.0186	0.9961	(0.64, 0.91)	196.98	1.15	0.9689

**Table 3:** Isotherm parameters for OTC adsorption onto willow residues at different temperatures.

Samples	T/K	ΔG/kJ·mol^−1^	ΔH/kJ·mol^−1^	ΔS/J·mol^−1^·K^−1^
WR-R	298	−10.28	37.49	160.29
	308	−11.88		
	318	−13.48		
WR-D	298	−14.35	39.18	179.63
	308	−16.15		
	318	−17.94		
WS-R	298	−10.21	33.05	145.18
	308	−11.67		
	318	−13.12		
WS-D	298	−11.76	31.68	145.76
	308	−13.21		
	318	−14.67		
WL-R	298	−9.51	38.36	160.63
	308	−10.91		
	318	−12.72		
WL-D	298	−11.95	20.47	108.79
	308	−13.04		
	318	−14.13		

**Table 4:** Elemental analysis, atomic ratios, and sugar content of willow residues.

Samples	Sugar (%)	N (%)	C (%)	H (%)	O (%)	H/C	(N + O)/C	O/C	K_d_ (L/kg)	K_oc_
WR-R	40.45	0.43	43.47	5.97	46.80	1.65	0.82	0.81	(49.42, 55.01)	119.46
WR-D	-	0.16	50.53	5.86	43.83	1.39	0.65	0.65	(196.42, 234.10)	432.91
WS-R	48.67	0.64	45.76	6.14	44.32	1.61	0.74	0.73	(38.88, 49.38)	95.15
WS-D	-	0.33	55.61	6.31	38.25	1.36	0.52	0.52	(129.36, 176.84)	272.09
WL-R	61.11	1.68	42.28	5.99	41.36	1.70	0.77	0.73	(37.66, 46.20)	94.39
WL-D	-	1.05	58.61	6.60	33.38	1.35	0.44	0.43	(124.34, 153.59)	234.04
